# Epigenetics may characterize asymptomatic COVID-19 infection

**DOI:** 10.1186/s40246-022-00401-3

**Published:** 2022-07-27

**Authors:** Cosby G. Arnold, Iain Konigsberg, Jason Y. Adams, Sunita Sharma, Neil Aggarwal, Andrew Hopkinson, Alexis Vest, Monica Campbell, Meher Boorgula, Ivana Yang, Christopher Gignoux, Kathleen C. Barnes, Andrew A. Monte

**Affiliations:** 1 Department of Emergency Medicine, University of Colorado – Anschutz Medical Campus, 12401 East 17th Avenue, 7th Floor, Aurora, CO 80045 USA; 2 Division of Biomedical Informatics and Personalized Medicine, University of Colorado – Anschutz Medical Campus, 13001 East 17th Place, Campus Box C290, Aurora, CO 80045 USA; 3 Division of Pulmonary, Critical Care and Sleep Medicine, University of California, Davis, 2835 J Street, Suite 400, Sacramento, CA 95816 USA; 4 Division of Pulmonary Sciences and Critical Care Medicine, University of Colorado – Anschutz Medical Campus, 12700 East 19th Avenue, Aurora, CO 9C0380045 USA; 5 Department of Emergency Medicine, University of California, Davis, Sacramento, USA

**Keywords:** SARS-CoV-2, COVID-19, Methylation, Epigenetics

## Abstract

RT-PCR is the foremost clinical test for diagnosis of COVID-19. Unfortunately, PCR-based testing has limitations and may not result in a positive test early in the course of infection before symptoms develop. Enveloped RNA viruses, such as coronaviruses, alter peripheral blood methylation and DNA methylation signatures may characterize asymptomatic versus symptomatic infection. We used Illumina’s Infinium MethylationEPIC BeadChip array to profile peripheral blood samples from 164 patients who tested positive for SARS-CoV-2 by RT-PCR, of whom 8 had no symptoms. Epigenome-wide association analysis identified 10 methylation sites associated with infection and a quantile–quantile plot showed little inflation. These preliminary results suggest that differences in methylation patterns may distinguish asymptomatic from symptomatic infection.

## Introduction

The severe acute respiratory syndrome coronavirus 2 (SARS-CoV-2) pandemic continues to represent an exceptional challenge for medicine and society. As of June 2022, there were more than 532 million confirmed cases worldwide with almost one fifth of these in the U.S.A [1]. Coronavirus disease 2019 (COVID-19), the disease caused by SARS-CoV-2, has a wide spectrum of severity with many infected individuals showing only mild or no symptoms [2]. The current testing strategy in the U.S. is limited primarily to symptomatic patients, an approach that fails to recognize the critical need for screening and surveillance. By waiting until patients progress to develop symptoms of COVID-19, we may be missing a critical intervention period. Understanding the epidemiology of asymptomatic and minimally symptomatic infection may have important implications for public health, including contact tracing, quarantine, and prediction of clinical outcomes.

Tests for SARS-CoV-2 infection based on reverse transcriptase polymerase chain reaction (RT-PCR) have limitations. PCR-based testing offers little diagnostic utility early after exposure, termed a “window period” after acquisition of infection [3]. Epigenetic markers reveal the underlying biology and can improve early diagnosis and risk stratification. Enveloped RNA viruses such as coronaviruses can manipulate the host’s epigenome via DNA methylation and thereby facilitate the identification of SARS-CoV-2 infected patients prior to symptom onset [4]. Moreover, these epigenetic patterns can differentiate patients who progress to severe disease [5]. This technology has the potential to optimize treatment and disposition decisions. In this study, we leveraged the Illumina Infinium MethylationEPIC BeadChip to evaluate DNA methylation patterns in asymptomatic and symptomatic SARS-CoV-2 infected patients.

## Materials and methods

We performed a case–control epigenome-wide association study (EWAS) to characterize asymptomatic versus symptomatic SARS-CoV-2 infection. This study was a sub-analysis of a larger study that described methylation patterns associated with COVID-19 severity, and the methods outlined here are as described previously [5].

All patients aged 18 years or older who presented to the University of Colorado Hospital from 1 March 2020 to 31 July 2020 and received a SARS-CoV-2 test by RT-PCR for any reason were eligible for this study (e.g., prior to hospitalization for COVID-19, surgical procedures, routine hospitalization, return to work clearance). Cases were defined as those who tested positive for SARS-CoV-2 by RT-PCR but had no symptoms; symptomatic patients with a positive test result served as controls. We tested peripheral blood DNA samples with the Illumina Infinium MethylationEPIC BeadChip and abstracted clinical data from the associated clinical visit from the electronic health record (EHR). All patients were followed across the University of Colorado Health (UCHealth) System over time for a minimum of 3 months to determine presence of symptoms at the time of the blood draw and to determine development and severity of symptoms and clinical outcomes (e.g., ED discharge, hospital admission, ICU, death).

DNA was extracted on the bead-based, automated extraction Maxwell® RSC System (Promega) and quantified using absorbance (NanoDrop 2000; Thermo Fisher Scientific, Waltham, MA) and fluorescence-based methods (Qubit; Thermo fisher Scientific, Waltham, MA). DNA quality was assessed with Agilent TapeStation (Agilent, Santa Clara, CA). Biospecimens were then uploaded to the Colorado Anschutz Research Genetics Organization (CARGO) laboratory information management system (LIMS).

Purified DNA samples were processed with Zymo EZ-96 DNA Methylation bisulfite conversion kits (Zymo, Irvine, CA). Random hexamer priming and Phi29 DNA polymerase were used for whole-genome amplification, and amplification products were enzymatically fragmented, purified from dNTPs, primers, and enzymes, and applied to the EPIC BeadChip.

EWAS analyses were performed on the entire epigenotyped dataset. Preprocessing and association testing was performed with the GLINT package. We used EPISTRUCTURE and ReFACTor to estimate components to adjust for population structure and to account for cell-type proportions, respectively. A linear mixed-effects model was fit to each probe and adjusted for age, sex, chip position, 6 ReFACTor components, 1 EPISTRUCTURE component, and a covariance matrix accounting for possible relatedness. Probes were annotated to CpG islands and genic regions with annotatr.

The study protocol was approved by the Colorado Multiple Institutional Review Board (COMIRB) and the research adheres to the ethical principles of research outlined in the U.S. Federal Policy for the Protection of Human Subjects. Patients were consented for blood collection and electronic health record (EHR) data abstraction through the University of Colorado COVID-19 Biorepository or the University of Colorado Emergency Medicine Specimen Biobank (EMSB). Raw data were generated at Health Data Compass and the Colorado Center for Personalized Medicine.

## Results

**Fig. 1:**
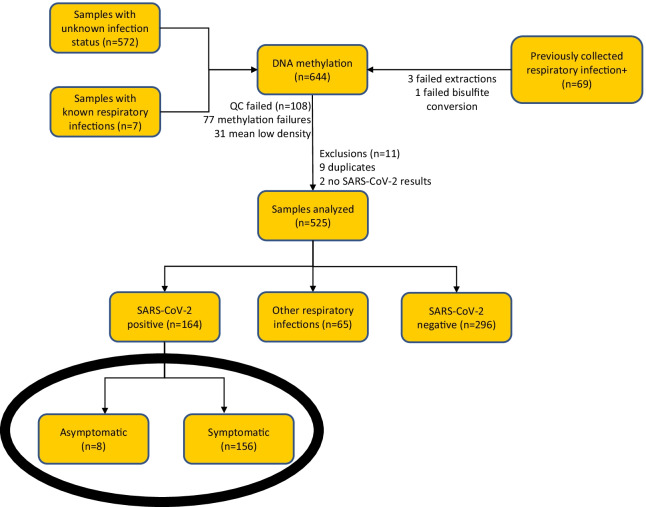
Study flow diagram

We enrolled 8 asymptomatic cases and 156 symptomatic controls (Fig. 1). None of the asymptomatic patients returned with symptoms during the follow-up period or had documentation of symptom development in the medical record during follow-up. Among the symptomatic patients, median age was 50 years, 88 (56%) were male, 22 (14%) Black, 43 (28%) White, and 85 (54%) Hispanic or Latino. Among asymptomatic patients, median age was 39.5 years, 5 (63%) were male, 3 (38%) Black, 3 (38%) White, and 3 (38% Hispanic or Latino). None of these demographic variables were statistically different between groups.

Analyses of clinical variables and disease severity are presented in Table 1. None of the asymptomatic patients required supplemental oxygenation or mechanical ventilation. Median overall oxygen saturation was higher in asymptomatic patients (*p* = 0.01) and this difference persisted in a sub-analysis of patients on room air (*p* = 0.004). The median oxygen flow rate among symptomatic patients requiring supplemental oxygen was 3 (IQR 2,5). Symptomatic patients had longer hospital stays and more frequently required intensive care unit (ICU) admission, although these observations did not reach statistical significance.

**Table 1:** Clinical characteristics of symptomatic and asymptomatic patients with COVID-19

	Symptomatic (*n* = 156)	Asymptomatic (*n* = 8)	*P* value
BMI	29.5 (25.4, 34.2)	35.7 (26.9, 37.4)	0.67
History of asthma	15 (9.68)	1 (12.50)	0.57
Heart rate	102.5 (90, 115)	94 (76, 127)	0.78
SpO2	92 (90, 95)	95.5 (94.5, 97)	0.01
Patients requiring supplemental oxygen	34 (24.46)	0 (0)	0.20
SpO2 in patients requiring supplemental oxygen	93 (90, 95)	NA	
SpO2 in patients on room air	92 (90, 95)	95.5 (94.5, 97)	0.004
Placed on ventilator	27 (17.53)	0 (0)	0.35
Admitted to ICU	43 (27.92)	1 (12.50)	0.68
ED disposition			0.58
Discharge	32 (20.65)	1 (12.50)	
Floor	101 (65.16)	7 (87.50)	
ICU	21 (13.55)	0 (0)	
Death	1 (0.65)	0 (0)	
Days in hospital	7 (3, 14)	5 (2, 6)	0.20
Final disposition			1
Home	114 (78.08)	7 (87.50)	
Nursing home or skilled rehabilitation	14 (9.59)	1 (12.50)	
Death	12 (8.22)	0 (0)	
Other	6 (4.11)	0 (0)	

Categorical variables, n (%); continuous variables, median (IQR)

*SpO2* oxygen saturation, *ICU* intensive care unit, *ED* emergency department

**Fig. 2:**
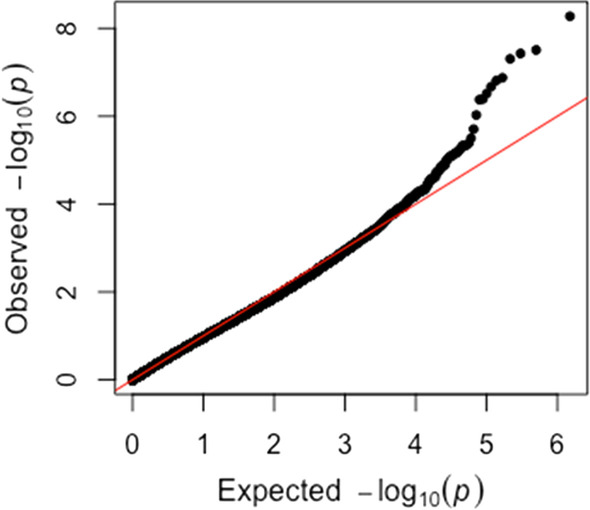
Quantile–quantile plot for the study cohort

We found clear signal genome-wide (after adjusting for ~ 750,000 tests), with 10 5′-C-phosphate-G-3′ nucleotides (CpGs) in immune and neurologic genes significant after false discovery rate (FDR) adjustment for multiple testing (Table 2). Furthermore, a quantile–quantile plot shows little inflation (as well as clear signal), with a lambda of 1.045257 (being close to 1, indicating the absence of systematic inflation of p-values, with only inflation at the tail for likely true signal) (Fig. 2). There is a clear pattern of methylation associated with asymptomatic SARS-CoV2 infected patients.

**Table 2:** Differential methylation patterns by gene location

Gene	Chromosomal location (hg19)	Gene function	Hypothetical clinical implications	*β*-coefficient	Difference in methylation (asymptomatic – symptomatic)	*Q *value*
F-box only protein 16 (FBXO16)	8.28302736	Promotes phosphorylation-dependent ubiquination and degradation, involved in controlling amount of protein in eukaryotes	Degradation of beta-catenin leads to decreased cell adherent protein expression, leading to disruption of cardiac electrical and mechanical communication between myocytes	− 0.23	− 0.07	0.004
Pleckstrin and Sec7 domain containing 3 (PSD3)	8.18663702	Codes protein, involved in endocytosis, implicated in tumor suppression	Increases signal transduction targeting proteins to subcellular compartments to respond to viral infection	− 0.14	− 0.13	0.009
Ribonucleic acid export 1 (RAE1)	20.55953214	Nucleocytoplasmic transport, mitotic checkpoint regulator	This tumor suppressor gene may be important for cell division in immune cells	-0.20	-0.11	0.009
NIPA like domain containing 2 (NIPAL2)	8.99306708	Autosomal recessive gene associated with congenital ichthyosis	Involved in transport of glucose and other sugars, likely in response to increased metabolic demands of infection	− 0.12	− 0.13	0.019
SH3 and multiple ankyrin repeat domains 2 (SHANK2)	11.70681592	Codes postsynaptic proteins at excitatory synapses, disruption may predispose to autism or developmental delay	May play a role in SARS-CoV-2 induced altered mental status	− 0.22	− 0.05	0.023
Phosphoglucomutase 1 (PGM1)	1.64117325	Codes isozyme of phosphoglucomutase (PGM), mutations cause glycogen storage disease type 14, highly polymorphic, mutations causes glycogen storage disease	Associated with breakdown and synthesis of glucose, upregulated during acute infection due to metabolic demands	− 0.15	− 0.11	0.029

*We report *q*-values rather than *p* values. *Q*-values are adjusted using the Benjamini–Hochberg Procedure, which controls the false discovery rate (FDR). The raw *p *values do not account for the number of statistical tests being performed and, without correcting for multiple tests, there would be many false positives due to chance

## Discussion

In the present study, we found that genome-wide patterns of DNA methylation differed in patients with asymptomatic and symptomatic infection with SARS-CoV-2. The genes identified as significant between asymptomatic versus symptomatic SARS-CoV-2 infected patients are expressed in a number of different tissues, from the skin to the central nervous system. Exactly how the differential expression of these genes interact to influence COVID-19 outcomes is unclear, although many have a role in immune function. A better understanding of COVID-19 epigenetics may help inform current disparities in COVID-19 and facilitate early, targeted interventions.

Prior studies of symptomatic patients have identified differentially methylated genes that distinguish SARS-CoV-2 infection from non-COVID-19 respiratory illness and that are associated with COVID-19 disease severity [5–7]. To our knowledge, our study is the first to evaluate methylation patterns in asymptomatic patients. While genetic studies in COVID-19 have identified gene clusters on chromosome 3 that are associated with disease severity, genetics alone do not adequately explain the underlying biology of COVID-19 with regard to disease severity [8]. If future studies substantiate our findings, epigenetics may prove helpful in predicting the host response to SARS-CoV-2 infection, both in asymptomatic and symptomatic patients, enabling risk stratification and personalization of both pre-exposure and early post-exposure therapies. As a compliment to genetic studies, epigenetic analyses may provide a link between environmental exposures, comorbidities, and infection with SARS-CoV-2 to enable a more complete understanding of the underlying biology.

Our analyses are limited by sample size and lack of follow-up outside the UCHealth System after the index visit. The UCHealth System cares for > 50% of Coloradoans across hundreds of clinics and 5 hospitals, but it remains possible that some asymptomatic patients could have developed mild symptoms without seeking care or been subsequently admitted with moderate-severe symptoms to a facility outside the UCHealth System. Additionally, our cohort includes viral samples taken early in the pandemic. It is possible that infections with strains such as Delta and Omicron might be associated with different host epigenetics and subsequent research should investigate whether the loci associated with asymptomatic illness in our study are strain specific or more broadly related to infection with SARS-CoV-2.

## Conclusions

Our findings suggest that DNA methylation is altered in asymptomatic SARS-CoV-2 infected patients relative to symptomatic patients. These preliminary findings are based on a small sample size; additional data are needed to determine whether our findings are generalizable and if the epigenetic signatures we observed in asymptomatic patients are predictive of eventual outcomes. Existing genetic studies have not sufficiently explained COVID-19 disease severity [8]. Epigenetic signatures provide a link between the host and environmental exposure, in this case SARS-CoV-2 infection, which leads to additional insights into host–pathogen interactions. An epigenetic methylation pattern that characterizes asymptomatic infection could help optimize the treatment of asymptomatic patients and reduce resource utilization, as these patients could be counseled that they will remain asymptomatic and do not require treatment or follow-up.

## Data Availability

De-identified data supporting the findings of this study are available from the corresponding author [CA] on request.

## Abbreviations

SARS-CoV-2: Severe acute respiratory syndrome coronavirus 2
COVID-19: Coronavirus disease 2019
RT-PCR: Reverse transcriptase polymerase chain reaction
EWAS: Epigenome-wide association study
EHR: Electronic health record
COMIRB: Colorado Multiple Institutional Review Board
EMSB: Emergency Medicine Specimen Biobank
FDR: False discovery rate
